# A Prognostic Marker in COVID-19 Disease Severity and Mortality: D-Dimer/Platelet Ratio

**DOI:** 10.7759/cureus.39580

**Published:** 2023-05-27

**Authors:** Alper Tahmaz, Aysegul Seremet Keskin, Filiz Kizilates

**Affiliations:** 1 Infectious Diseases and Clinical Microbiology, University of Health Sciences Antalya Training and Research Hospital, Antalya, TUR

**Keywords:** platelets, d-dimer, biomarker, in-hospital adverse outcomes, novel marker, d-dimer to platelets ratio, covid-19

## Abstract

Introduction: We aimed to examine the D-dimer/platelet ratio (DPR), which includes the combination of D-dimer and platelet measurements, which are two important markers in predicting prognosis, considering that it will show clinical progression.

Methods: After ranking the patients from high to low according to DPR level, they were divided into three equal groups. Demographic, clinical, and laboratory parameters between groups were compared according to DPR level. The consistency of DPR with other coronavirus disease 2019 (COVID-19) biomarkers in the literature in terms of hospitalization and mortality in the intensive care unit was examined.

Results: Complications such as renal failure, pulmonary thromboembolism (PTE), and stroke of the patients increased as the DPR increased. Patients in the third group with high DPR had higher oxygen demands from symptom onset, such as reservoir masks, high-flow oxygen, and mechanical ventilation. The first hospitalization location in the third group was determined as the intensive care unit. Mortality increased as the DPR value increased, and the time to death in patients in the third group was significantly shorter than the patients in the other two groups. While most of the patients in the first two groups recovered, 42% of the patients in the third group died. While the area under the curve was 80.6% in predicting DPR admission to the intensive care unit, the cut-off value was determined as 1.606. When the effect of DPR on predicting mortality was examined, the area under the curve for DPR was 82.6% and the cut-off value was determined as 2.284.

Conclusion: DPR is successful in predicting the severity, ICU admission, and mortality of COVID-19 patients.

## Introduction

Coronavirus disease 2019 (COVID-19) is a complex disease with a wide clinical spectrum from asymptomatic clinical presentation to severe respiratory failure resulting in multiple organ failure and death [1-3]. As part of immune function, infections trigger a complicated inflammatory reaction. The coagulation cascade is activated following the activation of the innate defensive systems. Cytokines that emerge in the inflammatory process also activate vascular endothelial cells and create a prothrombotic state by creating endothelial damage [4-6]. This is referred to as COVID-19-associated coagulopathy. It has been shown that bleeding does not develop in COVID-19, unlike disseminated intravascular coagulation (DIC) or sepsis-associated DIC. Coagulopathy appears to be connected to the extent of the illness [6]. Although its pathogenesis is not known yet, it is thought to occur as a result of "thrombo-inflammation". Microvascular thrombosis of the pulmonary vascular and other vascular structures is often reported in individuals infected with COVID-19 as a result of thrombo-inflammation activation. This contributes to organ failure and all-cause mortality [4-6].

D-dimer in plasma is a fibrin breakdown product. Coagulation, fibrinolysis, and thrombotic clots all raise D-dimer levels in the blood [7]. The D-dimer test, on the other hand, is not specific. Its levels rise in people suffering from heart attack, pneumonia, sepsis, malignancy, post-surgery, and pregnancy [7,8]. Furthermore, platelet activation and inflammation have been found to play a crucial role in the pathogenesis of COVID-19. Stimulation of platelets and reduction in platelet count due to cellular death can be used to indicate the severity and prognosis of COVID-19 [3].

Early and effective predictors of prognosis are needed for risk stratification of COVID-19 patients. In this work, we attempted to predict clinical progression in COVID-19 using the D-dimer/platelet ratio (DPR), which involves the combination of D-Dimer and platelet (PLT) measures, which are two essential indicators used to analyze the coagulation-fibrinolytic system in predicting prognosis.

## Materials and methods

This single-center cohort study was conducted in a tertiary hospital caring for COVID-19 patients. Before the study was conducted, approval was obtained from the Human Research Ethics Committee. Adult patients (i.e. patients aged 18 years and over) who applied to our hospital with the suspicion of COVID-19 and whose thorax computed tomography (CT) and/or polymerase chain reaction (PCR) results were conclusive, or a high probability of COVID-19 were analyzed. Diagnoses of COVID-19 were made according to World Health Organization interim guidance and confirmed by RNA detection of severe acute respiratory syndrome coronavirus 2 (SARS-CoV-2) by an onsite clinical laboratory. If a patient's PCR test was negative, the presence of clinical signs (fever of 38.3°C, cough, or shortness of breath) that could not be explained by any other disease or the presence of COVID-19 findings on thoracic CT caused the patient to be evaluated as a possible case of COVID-19.

We analyzed the data of 1922 patients who were hospitalized in the service or intensive care unit (ICU) for more than three days with the diagnosis of COVID-19 between 01-06-2021 and 31-01-2022. The list of patients diagnosed and examined with COVID-19 in the information technology department of the hospital was collected, and both patient files and imaging were reviewed retrospectively. Inclusion criteria were as follows: 18 years of age or older, diagnosis of COVID-19, and hospitalization for more than three days. Exclusion criteria included patients younger than 18 years of age, pregnant patients, and patients without data. Among 1922 patients, 1662 who met the study's criteria and whose data were complete were included in the study. The patients in the research were rated according to their DPR value, from the lowest to the greatest. Afterward, the patients were divided into three groups containing 554 patients in equal numbers. According to the DPR value, the lowest 1/3 segment refers to group 1, while the highest 1/3 segment refers to group 3. Demographic, clinical, and laboratory parameters between groups were compared according to DPR level. The consistency of DPR with other COVID-19 biomarkers in the literature in terms of hospitalization and mortality in the intensive care unit was examined. Platelet (hemogram; whole blood analysis) analysis was performed using the Sysmex XN-1000 (Sysmex, Kobe, Japan) system. D-Dimer assays were run on the ACL-TOP 700 (Instrumentation Laboratory, Bedford, Massachusetts, USA) coagulation autoanalyzer using commercial kits of ACL synthesis. The DPR was calculated by dividing the D-dimer (µg/L) level by the platelet (×10^3^/mm^3^) level.

The IBM Statistical Package for the Social Sciences (SPSS) for Windows 23.0 (IBM Corp., Armonk, NY) package application was used to analyze patient data obtained as part of the study. Descriptive values, frequency and percentage for categorical data, mean, and standard deviation for continuous data were provided. The "ANOVA test" was used to compare groups, while the "Chi-square or Fisher's Exact test" was used to analyze categorical data. The Post-Hoc Tukey test was performed to discover which groups were responsible for the significant difference in variables that exhibited a significant difference in the ANOVA test. ROC analysis was performed and the ROC curve was drawn for the parameters thought to have a distinctive effect on ICU admission and survival. A logistic regression analysis was also conducted to identify risk variables for ICU admission and survival. When the p-value was less than 0.05, the findings were considered statistically significant.

## Results

Of the 1922 patients whose COVID-19 reverse transcription polymerase chain reaction (RT-PCR) was positive or whose thoracic tomography was reported as COVID-19 pneumonia, 1662 patients with complete data were included in the study. They were divided into groups of 554 as low, medium, and high according to DPR value and evaluated. Out of the total, 57.3% (n=952) of the patients were male and there was no significant difference between the groups in terms of gender. The distribution of demographic and clinical findings by diagnosis groups is given in Table 1. There is a statistically significant difference between each of the diagnostic groups in terms of age and the presence of additional diseases such as heart failure, cerebrovascular accident (CVA), chronic obstructive pulmonary disease (COPD), diabetes mellitus (DM), kidney disease, leukemia lymphoma, and solid tumor (p<0.05). Patients in the third group with the highest DPR value were significantly older. Disease rates were higher in the third group in co-morbidities with a significant difference. Among the variables with significant differences in symptoms, fever and shortness of breath were higher in the third group with the highest DPR value. The saturation at the time of hospital admission was substantially lower in the third group compared to the other groups (p<0.05) (Table 1). 

**Table 1:** Investigation of demographic characteristics and symptoms of COVID-19 patient groups according to D-dimer/platelet ratio PAd = peripheral artery disease, CVA = cerebrovascular accident, COPD = chronic obstructive pulmonary disease, RT-PCR = reverse transcription polymerase chain reaction, * a = low, b = moderate, c = high, COVID-19 = coronavirus disease 2019

Variables (N=1662)	Low (0,006-0,82; n=554)	Moderate (0,82-1,79; n=554)	High (1,79-1410,2; n=554)	p-value	Difference*
	n (%) or Mean(SD)	n (%) or Mean(SD)	n (%) or Mean(SD)		
Age (years)	49 (15)	57(16)	64(16)	<0.001	a-b, a-c, b-c
Gender				0.430	
Male	329 (59.4)	315 (56.9)	308 (55.6)		
Female	225 (40.6)	239 (43.1)	246 (44.4)		
RT – PCR				<0.001	
Negative	4 (0.7)	20 (3.6)	108 (19.5)		
Positive	550 (99.3)	534 (96.4)	446 (80.5)		
Comorbid Diseases					
Myocardial infarction	3 (0.5)	3 (0.5)	2 (0.4)	0.882	
Heart Failure	8 (1.4)	14 (2.5)	24 (4.3)	0.012	
PAd	2 (0.4)	7 (1.3)	10 (1.8)	0.074	
CVA	4 (0.7)	12 (2.2)	22 (4)	0.001	
COPD	10 (1.8)	12 (2.2)	31 (5.6)	<0.001	
Diabetes	93 (16.8)	133 (24)	149 (26.9)	<0.001	
Renal Disease	7 (1.3)	21 (3.8)	43 (7.8)	<0.001	
Leukemia, Lymphoma	2 (0.4)	2 (0.4)	9 (1.6)	0.022	
Solid tumors	4 (0.7)	9 (1.6)	47 (8.5)	<0.001	
Symptoms					
Fever	115 (20.8)	121 (21.8)	156 (28.2)	0.007	
Fatique	185 (33.4)	182 (32.9)	132 (23.8)	<0.001	
Cough	266 (48)	280 (50.5)	211 (38.1)	<0.001	
Respiratory Distress	220 (39.7)	230 (41.5)	261 (47.1)	0.034	
Myalgia	104 (18.8)	96 (17.3)	72 (13)	0.026	
Sore throat	62 (11.2)	45 (8.1)	32 (5.8)	0.005	
Nausea	67 (12.1)	65 (11.7)	54 (9.7)	0.411	
Vomiting	41 (7.4)	35 (6.3)	33 (6)	0.600	
Chest Pain	32 (5.8)	21 (3.8)	28 (5.1)	0.299	
Back Pain	28 (5.1)	20 (3.6)	17 (3.1)	.212	
Loss of taste	49 (8.8)	24 (4.3)	19 (3.4)	<0.001	
Loss of smell	34 (6.1)	22 (4)	12 (2.2)	0.004	
Smoking	26 (4.7)	11 (2)	8 (1.4)	0.002	
Saturation at admission	95.8(4.4)	94.7(4.4)	93.9(5.3)	<0.001	a-b, a-c, b-c

According to lung tomography findings, the rate of individuals with lung involvement was higher in the second and third groups compared to the first group with low DPR values. When the laboratory parameters were compared, the alanine aminotransferase (ALT), aspartate aminotransferase (AST), lactate dehydrogenase (LDH), leukocyte, platelet, troponin, D-dimer, ferritin, and interleukin (IL)-6 levels of the patients in the third group were significantly higher than the patients in the other two groups (p<0.05), while lymphocytes were significantly lower (p<0.05). There was no difference between the patient groups in terms of neutrophil/lymphocyte ratio (Table 2). 

**Table 2:** Comparison of laboratory and tomography findings of COVID-19 patient groups according to DPR DPR = d-dimer platelet ratio, ALT = alanine aminotransferase, AST = aspartate aminotransferase, LDH = lactate dehydrogenase, CRP = C-reactive protein, WBC = white blood count, NLR = neutrophil/lymphocyte ratio, ESR = erythrocyte sedimentation rate, IL-6 = interleukin 6, COVID-19 = coronavirus disease 2019

Variables (N=1662)	Low (0,006-0,82; n=554)	Moderate (0,82-1,79; n=554)	High (1,79-1410,2; n=554)	p-value	Difference*
	n (%) or Mean(SD)	n (%) or Mean(SD)	n (%) or Mean(SD)		
Laboratory Findings					
ALT, U/L	34.7(33.3)	37.6(40.3)	56.8(143.6)	<0.001	a-c, b-c
AST, U/L	34.5(26.1)	43.6(35.7)	98.1(311.2)	<0.001	a-c, b-c
Albumin g/L	7.1(27.8)	8.9(40.1)	7.1(34.8)	0.720	
LDH, U/L	264.2(165.9)	325.9(237.7)	744.9(4538.4)	0.012	a-c
CRP, mg/L	45(59.5)	80.4(71.3)	124.5(102.3)	<0.001	a-b, a-c, b-c
WBC, × 10^3^/mm^3^	7.2(3.8)	7.2(4.1)	9.4(6.7)	<0.001	a-c, b-c
Hemoglobin, g/dL	13.3(1.9)	12.9(1.8)	11.9(2.2)	<0.001	a-b, a-c, b-c
Platelet, × 10^3^/mm^3^	260.5(129.9)	224(84.4)	195.5(98.1)	<0.001	a-b, a-c, b-c
Lymphocyte, × 10^3^/mm^3^	1.4(0.7)	1.2(0.6)	1.1(1.3)	<0.001	a-b, a-c
NLR	4.9(6.4)	11.3(102.1)	11(13.2)	0.132	
Troponin, ng/L	7.8(25.4)	17(64.1)	88.3(420.3)	<0.001	a-c, b-c
D-Dimer, ug/L	127.3(66)	275.8(122)	2262.5(5418.5)	<0.001	a-c, b-c
Fibrinogen, g/L	435.3(165.7)	506(175.4)	513.4(217.9)	<0.001	a-b, a-c
Ferritin, ng/mL	243.6(572.4)	345.8(448.4)	735(1918.1)	<0.001	a-c, b-c
ESR, mm/h	27.2(21.3)	36(20.8)	42.6(25.4)	<0.001	a-b, a-c, b-c
IL – 6, pg/mL	35.4(61.4)	147.8(577.9)	449(1060.8)	<0.001	a-c, b-c
Tomography				<0.001	
No involvement	155 (28)	97 (17.5)	128 (23.1)		
Unilateral involvement	63 (11.4)	50 (9)	64 (11.6)		
Bilateral involvement	336 (60.6)	407 (73.5)	362 (65.3)		

The most common complications were hyperglycemia (2.6%), acute renal failure (ARF) (2.5%), pulmonary thromboembolism (PTE) (2.2%), and stroke (1.7%). As the DPR value of the patients in terms of ARF, PTE, and stroke increased, the complication rate between the groups increased. Unlike the patients in the other two groups, the patients in the third group had higher oxygen needs from the onset of symptoms such as reservoir mask, high flow oxygen, continuous positive airway pressure (CPAP), and mechanical ventilation. While the first hospitalization location of the patients in the first two groups was significantly different in the form of ward hospitalization, the first hospitalization location of the patients in the third group was determined as the intensive care unit. The mortality rate increased as the DPR value increased, and the time to develop mortality was significantly shorter in the patients in the third group than in the patients in the other two groups. While most of the patients in the first two groups recovered (94% and 84.8%, respectively), a significant 42% of the patients in the third group died (Table 3). 

**Table 3:** Comparison of clinical progressions of COVID-19 patient groups by D-dimer/platelet ratio CPAP = continuous positive airway pressure, ICU = intensive care unit, MV = mechanical ventilation, ARDS = acute respiratory distress syndrome, PTE = pulmonary thromboembolism, GIS = gastrointestinal system, COVID-19 = coronavirus disease 2019

Variables (N=1662)	Low (0,006-0,82; n=554)	Moderate (0,82-1,79; n=554)	High (1,79-1410,2; n=554)	p-value	Difference^*^
	n (%) or Mean(SD)	n (%) or Mean(SD)	n (%) or Mean(SD)		
Oxygen requirement at first admission				<0.001	
Nasal Cannule	198 (71.5)	265 (66.4)	189 (42.3)		
Mask	35 (12.6)	65 (16.3)	78 (17.4)		
Mask with Reservoir	27 (9.7)	43 (10.8)	89 (19.9)		
High Flow	13 (4.7)	14 (3.5)	32 (7.2)		
CPAP	0 (0)	2 (0.5)	4 (0.9)		
Mechanical Ventilation	4 (1.4)	10 (2.5)	55 (12.3)		
Hospitalization at First Admission				<0.001	
Inpatient	545 (98.4)	524 (94.6)	440 (79.4)		
ICU	9 (1.6)	30 (5.4)	114 (20.6)		
Inpatient Hospitalization day	6.5(4.1)	7.6(4.8)	7.3(5.5)	<0.001	a-b, a-c
ICU duration day	12.2(10.8)	12.5(11.9)	8.9(7.5)	0.004	a-c, b-c
Total hospitalization day	7(5.7)	9(7.1)	9.6(7)	<0.001	a-b, a-c
Non ınvaziv MV days	4(1.4)	3.9(3.2)	3(1.5)	0.562	
Invaziv MV days	8(9)	7.8(7.7)	5.5(5.5)	0.018	b-c
Mortality	24 (4.3)	75 (13.5)	235 (42.4)	<0.001	
Mortality day	13.5(10.6	14.5(11.1)	10.7(8.2)	0.005	b-c
Days from the onset of symptoms to mortality	18(10.2)	19.1(10.8)	15.3(8.8)	0.007	b-c
Complications	30 (5.4)	64 (11.6)	94 (17)	<0.001	
ARDS	4 (0.7)	9 (1.6)	14 (2.5)	0.059	
Acute Renal Failure	5 (0.9)	11 (2)	26 (4.7)	<0.001	
PTE	6 (1.1)	8 (1.4)	23 (4.2)	<0.001	
Hyperglycemia	10 (1.8)	19 (3.4)	14 (2.5)	0.233	
Stroke	3 (0.5)	11 (2)	15 (2.7)	0.020	
GIS bleeding	4 (0.7)	9 (1.6)	6 (1.1)	0.364	
Final Status				<0.001	
Recovered	521 (94)	470 (84.8)	307 (55.4)		
Discharged with morbidity	9 (1.6)	9 (1.6)	12 (2.2)		
Mortalite	24 (4.3)	75 (13.5)	235 (42.4)		

According to the ROC analysis, in which DPR and other markers were examined, the area under the curve for C-reactive protein (CRP) was found to be 79.4%, while the cutoff value was determined as 90.3 in predicting the admission of the patients to the intensive care unit. For other parameters, the area under the curve and the limit values ​​are respectively determined to be 82.4% and 407 for D-dimer, 81.7% and 6.77 for neutrophil/lymphocyte ratio (NLR), 63.9% and 211.62 for platelet/lymphocyte ratio (PLR), 80.5% and 38.44 for CRP/albumin ratio (CAR), 80.6% and 1.606 for DPR. Since the diagnostic test evaluated in our study was staying in the intensive care unit, the value found for CRP was moderate (70-80%), for PLR weak (60-70%), for DPR, CAR, NLR, and D-dimer, it was determined that it had a good (80-90%) separation ability (Table 4). 

**Table 4:** ROC analysis result of laboratory parameters of ICU admission ROC = receiver operating characteristic, ICU = intensive care unit, CRP = C-reactive protein, NLR = neutrophil/lymphocyte ratio, PLR = platelet/lymphocyte ratio, CAR = CRP albumin ratio, DPR = D-dimer/platelet ratio

Risk Factor	AUC (95% CI)	Cut-off	P-value	Sensitivity (%)	Specificity (%)
CRP, mg/L	0.794 (0.763-0.825)	>90.3	<0.001	69.1	73.2
D-Dimer, µg/L	0.824 (0.796-0.853)	>407	<0.001	69.0	80.4
NLR	0.817 (0.788-0.847)	>6.77	<0.001	72.8	79.1
PLR	0.639 (0.598-0.680)	>211.62	<0.001	66.0	65.7
CAR	0.805 (0.775-0.836)	>38.44	<0.001	59.7	86.6
DPR	0.806 (0.776-0.835)	>1.606	<0.001	73.1	71.5

**Figure 1:**
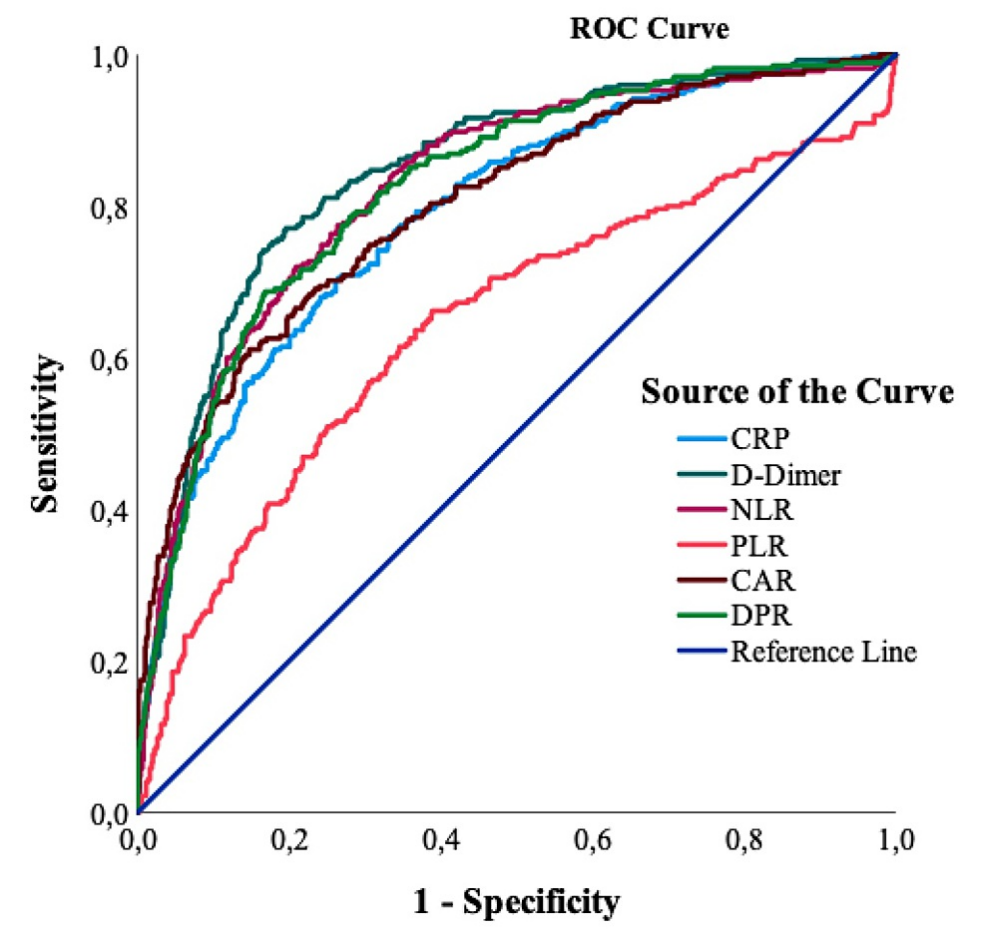
ROC analysis results on laboratory parameters of mortality ROC = receiver operating characteristic, CRP = C-reactive protein, NLR = neutrophil/lymphocyte ratio, PLR = platelet/lymphocyte ratio, CAR = CRP/albumin ratio and DPR = D-dimer/platelet ratio

According to the ROC analysis in which DPR and other markers were examined in predicting mortality in patients, the area under the curve for C-reactive protein (CRP) was found to be 79.3%, while the cutoff value was 91.9. For other parameters, the area under the curve and the limit values ​​are respectively; It was determined that it was 84.6% and 407 for D-Dimer, 82.8% and 6.53 for NLR, 64.3% and 211.62 for PLR, 80.3% and 37.25 for CAR, and 82.6% and 2.284 for DPR. Since the diagnostic test evaluated was the mortality of the patients, the value found for CRP was moderate (70-80%), poor level (60-70%) for PLR, good level (80-90%) for DPR, CAR, NLR, and D-dimer (80-90% separation ability was determined) (Table 5) (Figure 1). 

**Table 5:** ROC analysis results on laboratory parameters of mortality ROC = receiver operating characteristic, CRP = C-reactive protein, NLR = neutrophil/lymphocyte ratio, PLR = platelet/lymphocyte ratio, CAR = CRP/albumin ratio and DPR = D-dimer/platelet ratio

Risk Factor	AUC (95% CI)	Cut-off	P-value	Sensitivity (%)	Specificity (%)
CRP, mg/L	0.793 (0.762-0.824)	>91.9	<0.001	69.6	73.9
D-Dimer, µg/L	0.846 (0.819-0.873)	>407	<0.001	72.2	81.2
NLR	0.828 (0.799-0.856)	>6.53	<0.001	75.1	78.4
PLR	0.643 (0.602-0.684)	>211.62	<0.001	66.5	65.8
CAR	0.803 (0.772-0.834)	>37.25	<0.001	61.1	85.4
DPR	0.826 (0.798-0.855)	>2.284	<0.001	65.3	83.1

The distribution of risk factors affecting the admission to the intensive care unit of the patients included in the study is given in Table 6. In univariate analysis, age, presence of additional disease, fever, cough, shortness of breath, ALT, CRP, leukocytes, lymphocytes, NLR, D-dimer, IL-6 elevation, and uptake in tomography, PLR, CAR, and DPR values ​​increased intensely and were found to affect hospitalization. While it was determined that each unit increase in age increased admission to the intensive care unit 1.07 (95% CI, 1.06 to 1.08; p < 0.001) times, additional disease increased approximately 3 (95% CI, 2.3 to 3.79; p < 0.001) times, fever 2.5 (95% CI, 1.89 to 3.17; p < 0.001) times, shortness of breath 2.1 (95% CI, 1.69 to 2.75; p < 0.001) times increased admission to the intensive care unit. When the parameters that showed significant differences in univariate analysis were re-evaluated in multivariate analysis, increase in age, presence of fever and shortness of breath, increase in CRP, leukocyte and IL-6 counts were determined as factors that increased admission to the intensive care unit. 

**Table 6:** Evaluation of risk factors affecting ICU admission ICU = intensive care unit, ALT = alanine aminotransferase, LDH = lactate dehydrogenase, CRP = C-reactive protein, WBC = white blood count, NLR = neutrophil/lymphocyte ratio, IL-6 = interleukin 6, PLR = platelet/lymphocyte ratio, CAR = CRP/albumin ratio, DPR = D-dimer/platelet ratio

Variables	Univariant		Multivariant	
	Odds ratio (95% CI)	P-value	Odds ratio (95% CI)	p-value
Age (years)	1.07 (1.06-1.08)	< .001>	1.04 (1.02-1.07)	0.001
Gender				
Female	Reference	-		
Male	0.87 (0.68-1.11)	0.260		
Comorbidity	2.95 (2.30-3.79)	<0.001	1.10 (0.49-2.08)	0.986
Fever	2.45 (1.89-3.17)	<0.001	2.89 (1.39-6.05)	0.005
Cough	0.47 (0.36-0.60)	<0.001	0.92 (0.45-1.88)	0.822
Respiratory Distress	2.16 (1.69-2.75)	<0.001	1.89 (0.92-3.88)	0.085
Creatinine, mg/dL	1.10 (1.00-1.20)	0.673		
ALT, U/L	1.01 (1.00-1.05)	<0.001	1.00 (0.99-1.01)	0.711
LDH, U/L	1.00 (1.00-1.00)	0.273		
CRP, mg/L	1.012 (1.011-1.014)	<0.001	1.001 (.994-1.010)	<0.001
WBC, × 10^3^ / mm^3^	1.23 (1.19-1.26)	<0.001	1.18 (1.05-1.33)	0.005
Lymphocyte, × 10^3^ / mm^3^	0.55 (0.44-0.68)	<0.001	0.73 (0.37-1.41)	0.346
NLR	1.12 (1.10-1.13)	<0.001	1.01 (0.96-1.06)	0.726
D-Dimer, µg/L	1.01 (1.00-1.05)	<0.001	1.01 (1.00-1.05)	0.076
IL – 6, pg/mL	1.01 (1.00-1.05)	<0.001	1.01 (1.00-1.05)	0.046
Tomography	0.73 (0.56-0.96)	0.025	0.37 (0.14-1.02)	0.054
PLR	1.00 (1.002-1.004)	<0.001	1.00 (0.99-1.002)	0.881
CAR	1.04 (1.04-1.05)	<0.001	0.99 (0.96-1.03)	0.884
DPR	1.07 (1.05-1.09)	<0.001	1.01 (0.97-1.04)	0.534

The distribution of risk factors affecting the survival of the patients is given in Table 7. Survival in univariate analysis, age, comorbidity, fever, cough, shortness of breath, ALT, LDH, CRP, leukocytes, lymphocyte, NLR, D-dimer, IL-6 count, uptake on tomography, PLR, CAR, and DPR were determined to affect mortality. While it was determined that each increase in age increased the mortality 1.08 (95% CI, 1.07 to 1.09; p < 0.001) times, the presence of additional disease increased the mortality approximately 3 (95% CI, 2.48 to 4.09; p < 0.001) times, the presence of fever 2.7 (95% CI, 2.07 to 3.47; p < 0.001) times, and shortness of breath 2.1 (95% CI, 1.65 to 2.69; p < 0.001) times. When the parameters that showed significant differences in univariate analysis were re-evaluated in multivariate analysis, increased age, presence of fever and shortness of breath, and increased LDH and IL-6 numbers were determined as factors increasing mortality.

**Table 7:** Evaluation of risk factors affecting mortality ALT = alanine aminotransferase, LDH = lactate dehydrogenase, CRP = C-reactive protein, WBC = white blood count, NLR = neutrophil/lymphocyte Ratio, IL-6 = interleukin 6, PLR = platelet/lymphocyte ratio, CAR = CRP/albumin ratio, DPR = D-dimer/platelet ratio

Variables	Univariant		Multivariant	
	Odds ratio (95% CI)	p-value	Odds ratio (95% CI)	p-value
Age (years)	1.08 (1.07-1.09)	< .001>	1.06 (1.02-1.10)	< .001>
Gender				
Female	Reference	-		
Male	0.88 (0.69-1.12)	0.283		
Comorbidity	3.18 (2.48-4.09)	< .001>	1.28 (.53-3.10)	0.587
Fever	2.68 (2.07-3.47)	< .001>	3.22 (1.33-7.81)	0.009
Cough	0.44 (0.34-0.57)	< .001>	0.71 (0.30-1.70)	0.439
Respiratory Distress	2.11 (1.65-2.69)	< .001>	2.24 (0.92-5.49)	0.077
Creatinine, mg/dL	1.01 (1.0-1.02)	0.678		
ALT, U/L	1.006 (1.004-1.008)	< .001>	1.00 (0.99-1.01)	0.537
LDH, U/L	1.007 (1.006-1.008)	< .001>	1.006 (1.003-1.009)	< .001>
CRP, mg/L	1.012 (1.010-1.014)	< .001>	1.004 (1.000-1.010)	0.152
WBC, × 10^3^ / mm^3^	1.26 (1.22-1.31)	< .001>	1.07 (0.94-1.21)	0.308
Lymphocyte× 10^3^ / mm^3^	0.59 (0.48-0.73)	< .001>	0.76 (0.27-2.09)	0.588
NLR	1.12 (1.10-1.14)	< .001>	1.02 (0.96-1.07)	0.569
D-Dimer, µg/L	1.002 (1.000-1.004)	< .001>	1.00 (1.00-1.00)	0.314
IL – 6, pg/mL	1.014 1.009-1.019)	< .001>	1.008 (1.002-1.014)	0.011
Tomography	0.71 (0.54-0.94)	0.016	0.42 (0.11-1.66)	0.217
PLR	1.003 (1.002-1.003)	<0.001	1.00 (0.99-1.01)	0.468
CAR	1.042 (1.04-1.05)	<0.001	0.99 (0.95-1.04)	0.737
DPR	1.08 (1.06-1.10)	<0.001	1.01 (0.99-1.03)	0.429

## Discussion

Due to the heavy burden on the health system with increased cases of COVID-19 during the pandemic, there is a need for easy and reliable parameters that can be looked at during the evaluation of COVID-19 patients in the emergency room, predicting prognosis and mortality. In our study, we aimed to reveal the determinants of biochemical parameters that may affect the need for treatment and mortality in the intensive care unit of inpatients with the diagnosis of COVID-19 and to examine the relationship of DPR with other parameters and clinics as a new marker. Although there are many studies in the field of demonstrating mortality and prognosis, it is the first study on DPR.

In the literature, DPR was used as a new marker of risk classification in patients diagnosed with PTE, and it was observed that the PTE clinic was worse in patients with increased DPR compared to other patients [7]. In another study, increased DPR was found to be significant in differentiating preeclampsia from normal pregnancy and gestational hypertension [9]. Our study concluded that the DPR value is an effective marker in showing the prognosis and mortality in patients with COVID-19. Since group 3 patients with high DPR had higher oxygen needs than patients in other groups, their first hospitalization was ICU, and they were more mortal.

Age is one of the most important prognostic markers commonly known in COVID-19 patients. Advanced age is associated with poor outcomes in terms of death, hospitalization, and ICU admission [10,11]. The patients in group 3 with poor clinical progression were significantly older than the other two groups. It was thought that it may be due to reasons such as the weakening of the immune system with aging, an increase in comorbid diseases, and a decrease in organ functions.

Studies show that lymphopenia and high NLR values may be prognostic factors and markers of mortality [12,13], and hemoglobin levels are lower in severe disease [14,15]. Wang et al. found that thrombocyte levels were lower in patients needing intensive care unit than in ward patients [16]. Herold et al. found that high CRP values ​​were associated with severe disease and respiratory failure [17], and Velevan et al. found that high CRP levels were associated with mortality [18]. Gao et al., on the other hand, found higher D-dimer levels in the group with severe disease [19]. In a meta-analysis including 19 studies, LDH levels were found to be higher in the critically ill group [20], and in the studies conducted by Mehta et al., ferritin levels were found to be significantly higher in the group of patients who died due to COVID-19 [21]. Del Valle et al. found an increase in IL-6 levels as an independent risk factor that predicts prognosis and mortality [22]. Kalyon et al. found CAR values ​​valuable in predicting mortality and prognosis in elderly patients [23].

In our study, when the cut-off value for DPR was taken as 1.606 in predicting ICU admission, the AUC was 0.806, and it was a good predictor (80-90%) for DPR, CAR, NLR, and D-dimer. When the cut-off value for DPR was taken as 2.284 to predict mortality, the AUC was 0.826, and it was a good (80-90%) predictive marker for DPR, CAR, NLR, and D-dimer. In our study, a significant increase in LDH, CRP, leukocyte, D-dimer, ferritin, fibrinogen, and IL-6 levels, and a decrease in hemoglobin, thrombocyte, and lymphocyte levels were observed among the groups, in accordance with the literature, together with the increase in the DPR rate between the groups. According to the literature, an increase in D-dimer and a decrease in platelets in severe patient profiles in COVID-19 is a known reality, and we expected the DPR obtained as a result of the joint use of these two parameters to be better than other prognostic markers and ratios in the literature. However, in our study, D-dimer and NLR were found to be better than DPR in predicting ICU admission and mortality. This may be due to the fact that some patients received oral steroid therapy during outpatient follow-up to reduce hospitalizations during the COVID-19 pandemic. Therefore, although we could not see the decrease in platelet level as we expected, neutrophilia may have occurred. Due to the retrospective nature of our study and the lack of clear information on steroid use in patient files, we could not differentiate this factor clearly in our patients. This was one of the shortcomings of our study.

In the univariate analysis of clinical findings and laboratory parameters that differed between the groups, it was seen that many reasons increased ICU admission and mortality. ICU admission was determined that the presence of comorbidity increased three times, the presence of fever 2.5 times, and shortness of breath 2.2 times. The presence of comorbidity increased 3.2 times, the presence of fever 2.7 times, and shortness of breath 2.1 times in mortality. In multivariate analysis, increased age, presence of fever and shortness of breath, and increase in CRP, leukocytes, and IL-6 levels were determined as independent predictors of ICU admission, while independent predictors of mortality were increased age, presence of fever and shortness of breath, and increase in leukocytes and IL-6 levels observed. The significantly higher age, comorbidities, fever, and dyspnea symptoms of group 3 patients with high DPR make DPR valuable in terms of demonstrating the progress and mortality of the patients.

The increase in the DPR rate and the significant difference in complications between the groups were important in terms of demonstrating clinical prognosis. A significant increase was observed in ARF, PTE, and stroke in coagulopathy-related complications, especially among the groups, with an increase in DPR.

A study by Rashed found that 21.8% of 952 hospitalized COVID-19 patients died, while Iskit found a death rate of 32% in COVID-19 patients hospitalized in intensive care [24,25]. In our study, the mortality rate was 20.1% in all patients, while the mortality rate was 42.4% in group 3 patients with high intensive care admissions. We think that the reason for the high mortality rate in our study is that it was performed in a training and research hospital. Because in our country, we see the clinical situation as milder COVID-19 cases in provincial and district state hospitals, and the treatment of COVID-19 patients needing ICU whose clinical picture is more severe in city hospitals and university hospitals.

However, some limitations of this study should be mentioned. This study was a single-center retrospective study. Thus, the possibility of missing asymptomatic or mild patients, the inability to monitor the changes in parameters in these patients due to the single blood result of patients whose clinical exacerbation rapidly exacerbated, and the uncertainty of the effect of patient treatment on blood results were the limitations of the study. Another limitation was that our study did not include the pediatric age group and pregnant women.

## Conclusions

Understanding the course of the disease is of paramount importance when analyzing patients suspected of having COVID-19 due to SARS-CoV-2 infection, which has resulted in high morbidity-mortality rates and increased need for ICU admission. DPR can be computed fast, simply, and cheaply, even during the initial hospitalization. It is highly accurate in estimating the severity, ICU admissions, and mortality rates of COVID-19 patients. As a result, these factors may be computed immediately after admission and utilized to forecast the clinical course of COVID-19 patients.
